# Penza's Surgical Maneuver: A Key to Tackle Complex Proximal Descending Thoracic Aortic Infectious or Malignant Issues

**DOI:** 10.1055/s-0040-1714059

**Published:** 2020-11-05

**Authors:** Evgeny Rosseykin, Dmitry Chichevatov, Evgeny Sinev, Evgeny Kobzev

**Affiliations:** 1 Department of Surgery, Federal Center for Cardiovascular Surgery, Penza, Russian Federation; 2 Department of Surgery, Penza State University, Penza, Russian Federation; 3 Department of Thoracic Surgery, Regional Oncology Health Center, Penza, Russian Federation

**Keywords:** thoracic aorta, cardiopulmonary bypass, vascular surgical procedure, esophagoplasty, thoracic malignancies

## Abstract

We performed off-pump ascending-to-descending aortic grafting with the debranching of left carotid and subclavian arteries and total aortic arch transection in three patients. We have called this technique “Penza's Surgical Maneuver” and propose it for a safe surgery in cases where the aortic arch, the aortic isthmus, the beginning segment of the descending aorta, the esophagus, and a lung are diseased.

## Introduction

Simultaneous thoracic aorta, lung, and esophagus resection remain quite a rare procedure, mainly because of the high technical complexity of the surgery and the need for cardiopulmonary bypass (CPB) or extracorporeal membrane oxygenation. 1 Most articles are dedicated to concomitant lung resection surgeries and anatomic aortic replacement. 2 3 4

We propose a unified surgical approach we have called “Penza's Surgical Maneuver (PSM).” The main purpose of this technique is to prepare an aortic segment that is safe for surgery and includes the following: (1) aortic arch, (2) aortic isthmus, and (3) beginning segment of descending aorta. We have called such a segment “defunctionalized aortic zone” (DAZ).

## Technique

**Fig. 1:**
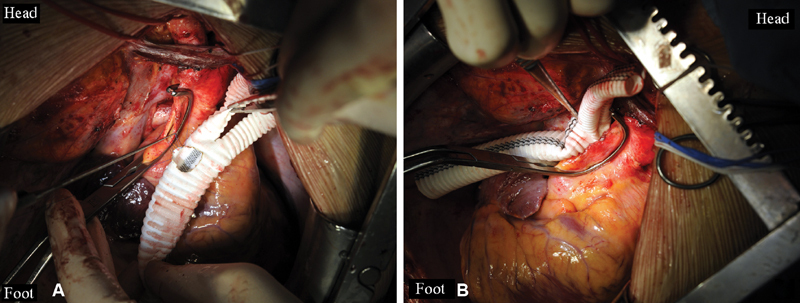
The side-to-side anastomosis of the bifurcation graft to the ascending aorta: ( **A** ) the running suture, polypropylene 5–0; ( **B** ) the final view.

**Fig. 2:**
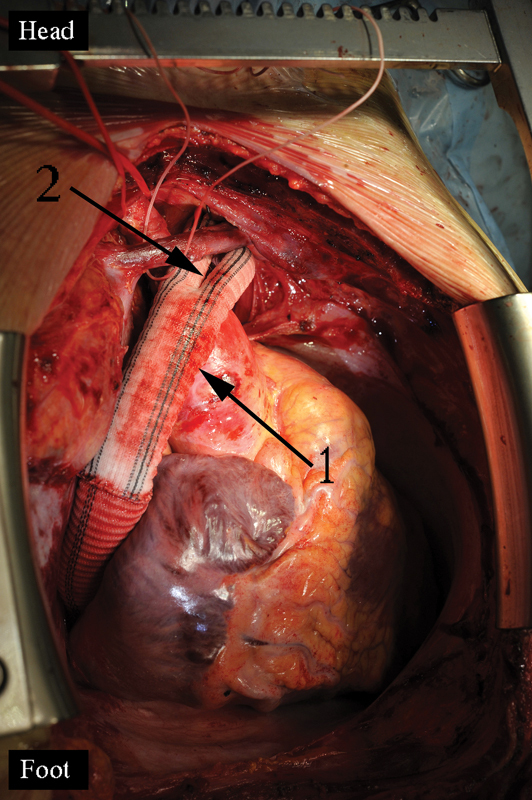
The end-to-end anastomoses of the left carotid and subclavian arteries to the graft branches: 1, zone of the ascending aorta to the graft anastomosis; 2, upward to the neck anastomosed branches.

**Fig. 3:**
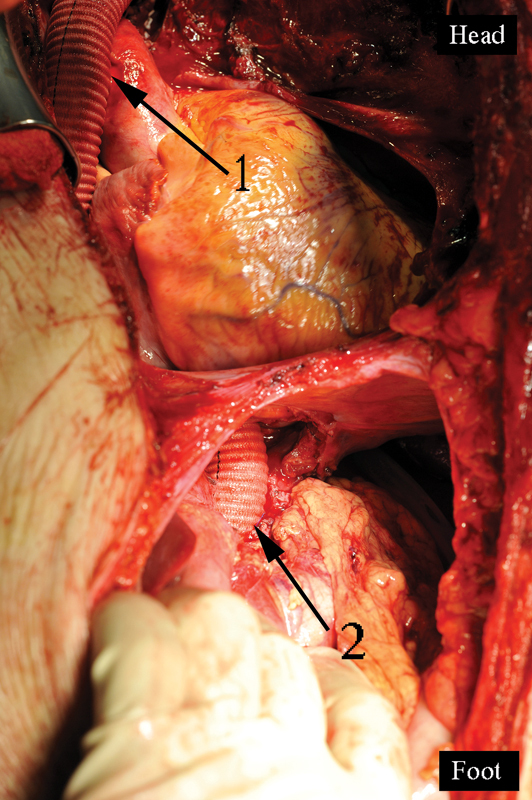
The sight of the two grafts to the aorta anastomoses: 1, zone of the ascending aorta to the bifurcation graft anastomosis; 2, zone of the end-to-side anastomosis of the linear graft to the abdominal epigastric aorta.

**Fig. 4:**
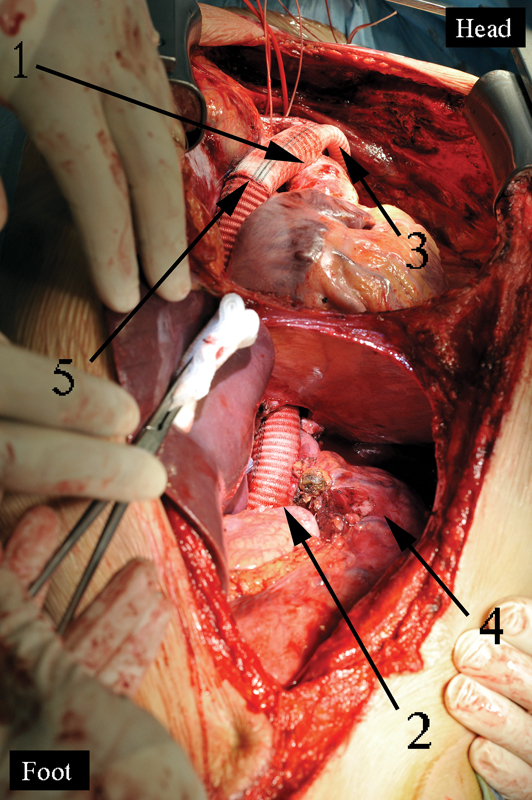
View of the completed bypass: 1, the ascending aorta to the bifurcation graft side-to-side anastomosis; 2, the end-to-side anastomosis of the linear graft to the abdominal epigastric aorta; 3, the upward left branch anastomosed to the left subclavian artery; 4, the stomach prepared for the Ivor–Lewis esophagoplasty; 5, the end-to-end anastomosis of the linear graft to the bifurcation graft.

**Fig. 5:**
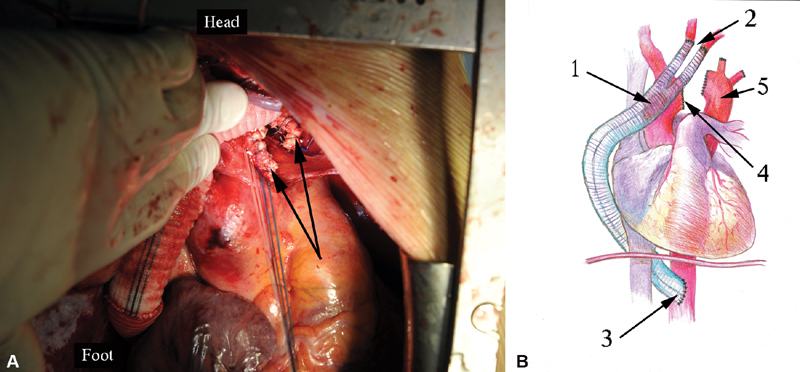
The final stage of Penza's surgical maneuver (PSM). Aortic arch transection ( **A** ) arrows point to the proximal and distal aortic stumps. ( **B** ) The scheme of complete PSM: 1, the ascending aorta to the bifurcation graft anastomosis; 2, debranched left subclavian and carotid arteries; 3, the anastomosis of the linear graft to the abdominal epigastric aorta; 4, transected aortic arch; 5, defunctionalized aortic zone.

PSM steps are described below:

Step 1: median sternotomy and contiguous upper median laparotomy are performed. Cardiac surgery begins with off-pump ascending-to-descending aortic grafting. The bifurcation graft is anastomosed side-to-side to the ascending aorta ( Fig. 1 ).
Step 2: left carotid and subclavian arteries are debranched into the aortic graft with an end-to-end anastomoses ( Fig. 2 ). The purpose of the debranching is to create a free aortic arch zone for further transection.
Step 3: the other linear aortic graft is guided intrapericardially, retrocavally, and transdiaphragmally and anastomosed end-to-side to the epigastric segment of the abdominal aorta ( Fig. 3 ). The general thoracic surgery team, simultaneously operating in the laparotomy area, performs any required gastric preparations. If concomitant esophagoplasty is planned, then a gastroepiploic transplant is prepared. In other cases, it is only necessary to prepare an epiploic transplant to use as a sealant biomaterial in the mediastinum.
Step 4: the bifurcated (from ascending aorta) and linear grafts (from abdominal aorta) are now connected end to end ( Fig. 4 ).
Step 5: the aortic arch is transected and tightly stitched immediately distal to the brachiocephalic trunk. Thus, we create a DAZ that is shut off from the antegrade circulation ( Fig. 5 ).
Step 6: the sternolaparotomic wound is closed after the mediastinum and the abdomen have been drained. From any thoracotomy, DAZ can be resected at any level.

## Clinical Experience

**Fig. 6:**
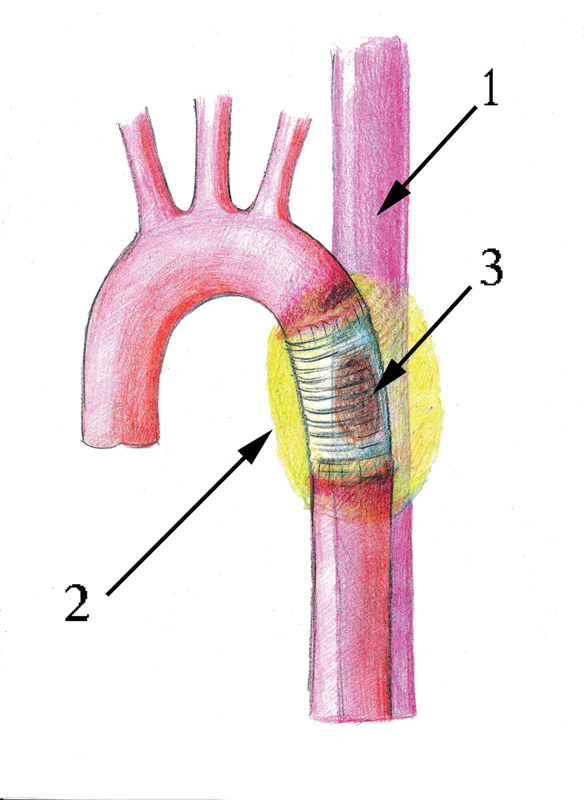
Schematic illustration of the disease in patient 1: 1, the esophagus; 2, the paraprosthetic pyogenic cavity; 3, the fistula connecting the paraprosthetic cavity to the esophagus.

Patient 1, a 28-year-old male, intravenous drug-dependent patient, presented with an infected aortic graft with recurrent pulmonary bleeding and esophagus fistula. Five years before, he underwent thoracic aorta resection and replacement with a synthetic graft due to aortic coarctation. Over the 5 years, the graft became infected and a paraprosthetic cavity connected to the esophagus with a fistula of 8 mm in diameter was formed ( Fig. 6 ). Additionally, there were multiple fistulas connecting the paraprosthetic cavity to the left lung parenchyma, which allowed for pulmonary bleeding.

**Fig. 7:**
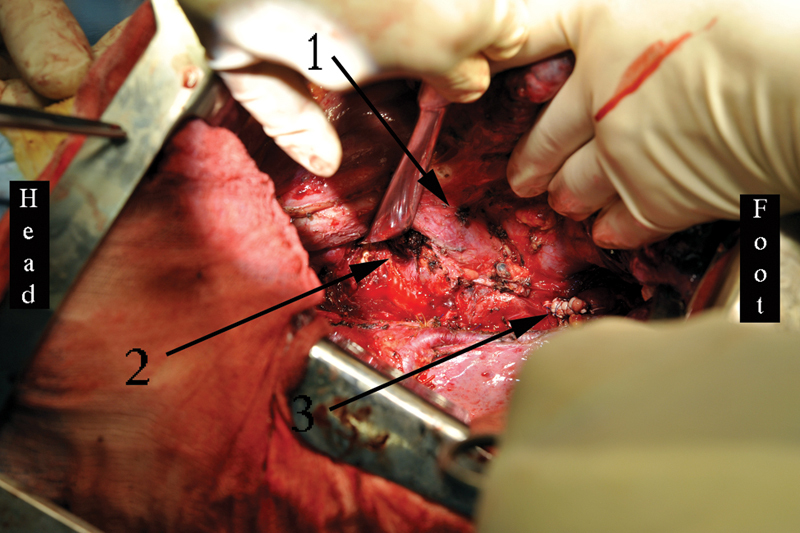
Right-sided thoracotomy. Defunctionalized aortic zone (DAZ) and the esophagus are resected: 1, tracheal bifurcation; 2, zone of the left lung with fistulas that have to be sealed; 3, the distal aortic stump after DAZ resection.

The patient underwent the following 11-hour surgical procedure. After the PSM and gastric mobilization were finished, a right-sided thoracotomy was performed. The esophagus was mobilized and transected 2-cm proximal to the fistula. An en block resection of the upper part of the DAZ, including the graft, the infected paraprosthetic fibrous capsule, and the esophagus was done ( Fig. 7 ). The pulmonary fistulas were stitched with reverse U-shaped pledget sutures. Ivor–Lewis type esophagoplasty was performed. The zone of the paraprosthetic cavity and lung fistulas was filled with the omentum.

**Fig. 8:**
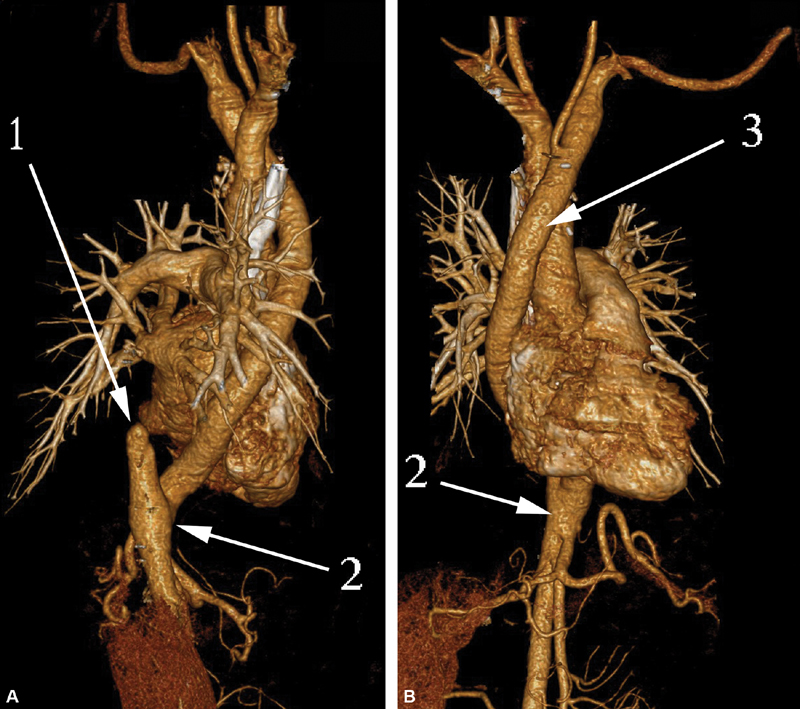
Computed tomography volume rendering lateral view ( **A** ) and frontal view ( **B** ) of patient 1. Seven years after surgery: 1, the distal aortic stump, 2, the graft to the abdominal aorta anastomosis, 3, the graft to the ascending aorta anastomosis.

The postoperative period was complicated by stroke causing right-sided hemiparesis. The patient was transferred to the neurology department on the postoperative day 12. The patient was followed up for 7 years ( Fig. 8 ). Neurology deficit is completely resolved, and patient has returned to his work and stop narcotic use.

**Fig. 9:**
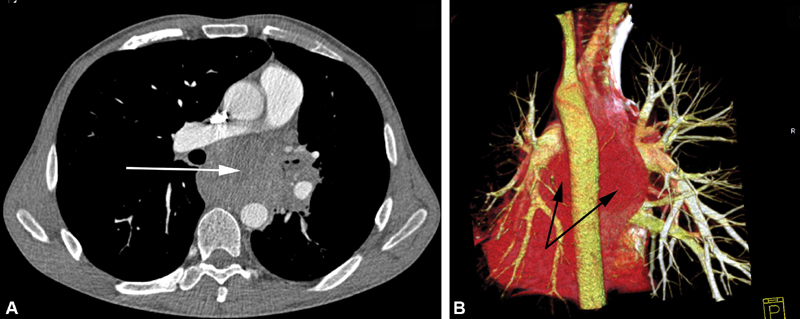
Computed tomography (CT) scan ( **A** ) and CT volume rendering ( **B** ) of patient 2. Arrows point to the tumor mass.

**Fig. 10:**
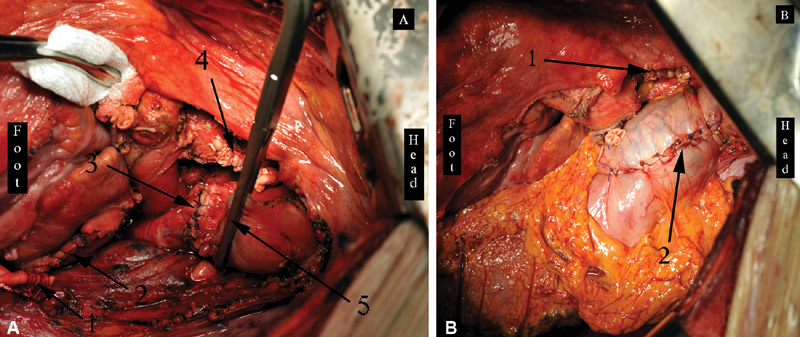
Stages of the operation via the left-sided thoracotomy with patient 2. ( **A** ) Left-sided pneumonectomy with the pericardium, left atrium, defunctionalized aortic zone, and esophagus resection: 1, the distal aortic stump; 2, the running suture of the left atrium; 3, the bronchial stump; 4, the proximal aortic (aortic arch) stump; 5, the clumped esophageal stump. ( **B** ) The final view of the operation: 1, the proximal aortic (aortic arch) stump; 2, the left-sided Ivor–Lewis esophagoplasty with the hand-made esophagogastric anastomosis.

Patient 2, a 44-year-old male presented with left-sided pulmonary central adenocarcinoma, T3N0M0, and midthoracic esophageal squamous cell cancer, T3N1M0, with para-aortic metastatic invasion into the thoracic aorta ( Fig. 9 ). He underwent surgery that lasted 10 hours. After PSM and gastric mobilization were finished, left-sided thoracotomy and combined pneumonectomy with esophageal and DAZ, resection to the inferior pulmonary vein level were performed. Ivor–Lewis type esophagoplasty was done ( Fig. 10 ).

**Fig. 11:**
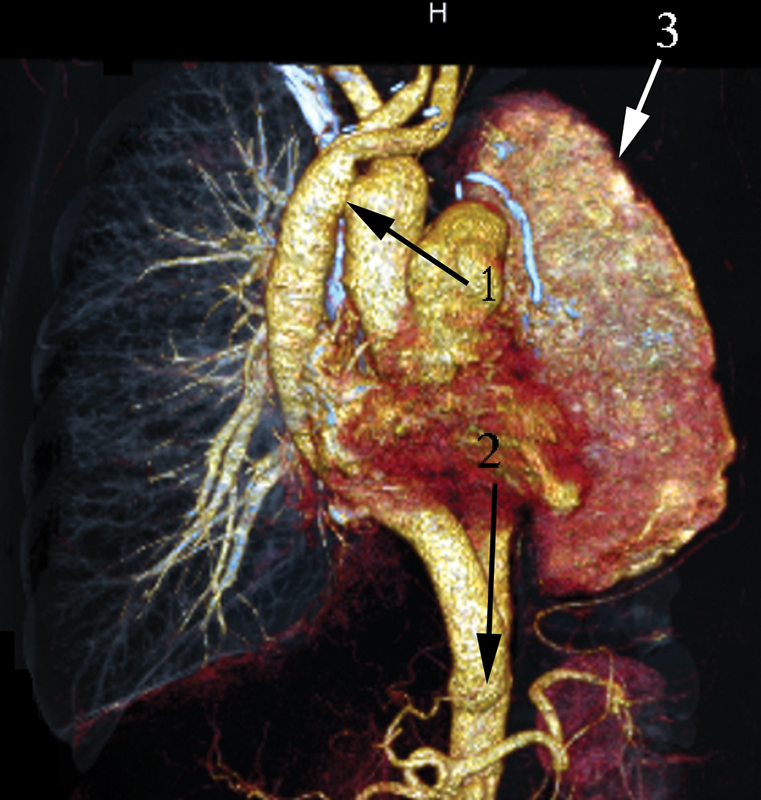
Computed tomography volume rendering of patient 2. Forty months past surgery: 1, the ascending aorta to the graft side-to-side anastomosis; 2, the end-to-side anastomosis of the graft to the abdominal epigastric aorta; 3, the stomach in the vast empty pleural cavity.

The postoperative period was uneventful. The patient was discharged on postoperative day 14. Three courses of adjuvant chemotherapy were performed. The patient was followed up for 6, 12, 18, 24, 32, and 40 months ( Fig. 11 ). Radiological computed tomography (CT) follow-up did not reveal any signs of recurrent tumors in the tumor bed. The patient's condition was stable with very good social activity. CT after 40 months disclosed numerous metastases. The patient died 44 months after our operation due to lung cancer progression.

**Fig. 12:**
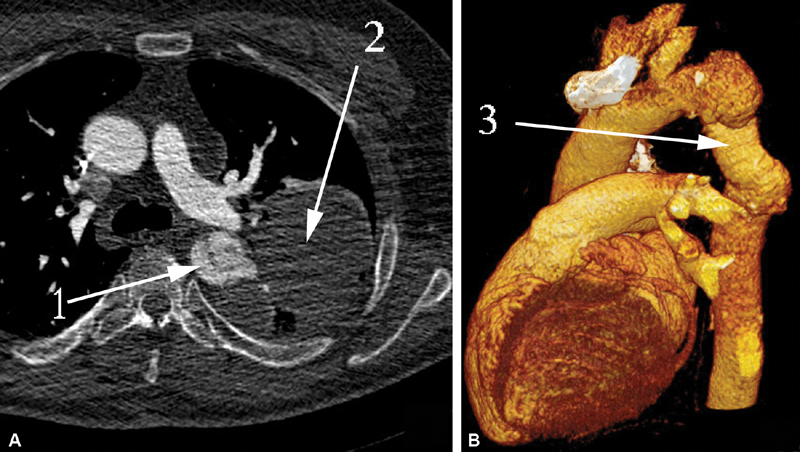
Computed tomography (CT) scan ( **A** ) and CT volume rendering ( **B** ) of patient 3: 1, zone of the aortic graft pseudoaneurysm; 2, the hemothorax mass; 3, the prosthesis.

Patient 3, a 56-year–old male, 35 years after thoracic aorta grafting due to aortic coarctation, presented with the rupture of aortic graft pseudoaneurysm, which caused hemothorax and pulmonary bleeding ( Fig. 12 ).

**Fig. 13:**
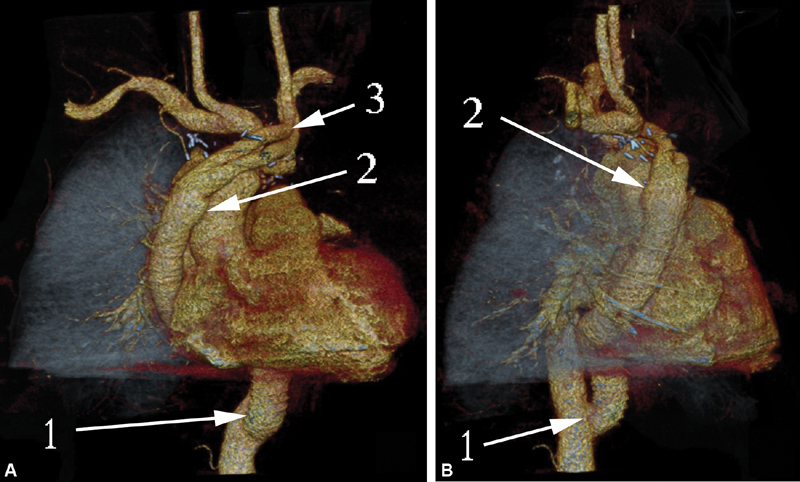
Computed tomography volume rendering frontal view ( **A** ) and lateral view ( **B** ) of patient 3. Third postoperative day: 1, the anastomosis of the graft to the abdominal epigastric aorta; 2, the ascending aorta to the graft anastomosis; 3, debranched left subclavian and carotid arteries.

He underwent an 11-hour emergency surgery. After PSM, DAZ (including “old” graft and aneurysm) resection through left-sided thoracotomy was also done. Because of the left-lung destruction caused by extensive parenchymal hematoma, pneumonectomy was performed ( Fig. 13 ).

The postoperative period was complicated by severe pulmonary and cardiac failure. Intensive care unit stay lasted for 26 days. The patient was discharged on postoperative day 33. After 35 months of follow-up, the patient's condition is stable, he is mobile, and his complete social adaptation has been achieved.

## Discussion

We have named the surgical technique described above “maneuver” because we consider it as a complete, undivided, conceptual procedure that is sure to include an approach, off-pump ascending-to-descending aortic grafting, debranching of two left arch branches, and an aortic arch transection. While every item is well-known, we have never met an intention to join them into an essential procedure. To emphasize this, we have permitted ourselves the proper name “Penza surgical maneuver.”

We think that the PSM has several advantages, including no need in CPB and circulatory arrest, which are important to maintain hemostasis during further surgery stages, and performance in an anatomically intact region, which facilitates surgical manipulations.

Aortic arch and aortic isthmus surgery is complicated, unsafe, and ambiguous in terms of approaches, even with CPB. 4 Cervical end-to-side debranching of the left subclavian artery into the left carotid artery with the further on-pump intrathoracic orthotopic vascular interventions has been proposed as a technical approach to solve this problem. 5 We believe that PSM and DAZ creations make all thoracic aorta segments approachable from any thoracotomy and render aortic resection much easier and safer, as there is no need to mobilize the aortic arch and the aortic isthmus through thoracotomy.

The first side-to-side anastomosis between the ascending aorta and the bifurcation graft is left open from both sides, which, we feel, permits anatomically correct graft positioning at both ends later in the case. We anastomosed the graft’s distal end to the epigastric segment of aorta as it was described by Wukasch et al. 6 Such an approach is especially reasonable in concomitant esophagoplasty, since the access to the mentioned aortic segment is facilitated when esophageal and gastric mobilization is done. Right sided crurotomy, sagittal diaphragmotomy, periaortic fat, and lymph node excision are the common procedures in lymph node dissection for esophageal cancer. We have always managed to mobilize at least 10 cm of the anterolateral wall of the epigastric aorta, which was enough to form an anastomosis. Moreover, it is technically much easier to create a vascular anastomosis with the epigastric aortic segment under the diaphragm through sternolaparotomy than with the thoracic aorta. In this case, there is no need to rotate the heart cranially, which could require CPB. The latter is especially important when the heart is dilated. 7 Besides, forming an anastomosis with the epigastric aortic segment allows keeping the pleural cavity intact in case of pleural infection, avoiding lung injury if there are severe pleural adhesions, and preventing dangerous transthoracic manipulations in the presence of intrapleural bleeding. Stitching the graft's distal end to the epigastric aortic segment permits forming a more physiological anastomosis at acute angle compared with the anastomosis with the thoracic aorta, where a right angle is created.

The clear-cut advantage of the PSM is that it allows a graft to be fully isolated within the pericardium and the abdomen, which prevents its contact with the area of the main pathological process. This is highly important when the consequent lung, esophagus, or thoracic aorta surgery carries a risk for the graft to become infected.

When carrying out concomitant esophageal resection, we preferred performing primary esophagoplasty. Since sternolaparotomy is done by two surgical teams working simultaneously, gastric transplant preparation does not take additional time. We have always finished gastric mobilization before the PSM is done. The quality of life of a patient after primary esophagoplasty is, undoubtedly, higher. Delayed restoration of gastrointestinal continuity after PSM with esophageal and DAZ resection seems to be difficult. The distinct advantage of concomitant esophagoplasty permits the use of the transposed omentum as a plastic material with unique biological properties. This matters especially when there is a risk for the aortic graft to be infected.

DAZ allows us to choose either side to perform the required thoracotomy. The aorta with the transected aortic arch becomes accessible from both sides to safely conduct the surgery. In the first presented case, we chose right-sided thoracotomy to resect the thoracic aorta, since we did not want to do a traumatic left-sided pneumolysis and take down connective-tissue adhesions. The right-sided approach was performed through the intact pleural cavity in comfortable anatomical conditions. Keeping the left lung safe and plugging the infected graft area with the omentum let us immediately achieve complete aerostasis avoidance of air leak and prevention of infection dissemination. In the second and third cases, the benefits of left-sided thoracotomy are clear. Creation of DAZ facilitated the left-sided Ivor–Lewis procedure in patient 2.

The obvious disadvantages of PSM are the need for three different surgical approaches and the long surgery duration. It is notable that 10 to 11 hours of operation are a total time including either DAZ preparation or difficult resection and reconstruction. Thereafter, we may not estimate whether surgery duration decreased under the conditions of CPB. Probably these changes might be insignificant. Sternotomy and continuing upper laparotomy, anastomosing of the prosthesis to the epigastric segment of an aorta give an ability to reduce wound length, and prepare gastric and omental transplants perfectly well without an additional time. This may be considered as an advantage of our technique. We suppose thoracotomy to be obviously essential. The steps sequence described above meets principles of patient's safety, an efficient surgical control during resection of the aorta, and a proper hemostasis.

CPB is a routine and harmless procedure nowadays. But all our patients underwent a complex surgery in the area of the aortic arch and isthmus. So, CPB has to be used with hypothermia and circulatory arrest. 8 Such approach cannot be considered very safe in the context of brain and visceral organs protection. Moreover, hemostasis may be very doubtful in this scenario.

Some authors reported different techniques that may be positioned as alternatives to PSM. Arakelyan et al 9 proposed ascending-to-descending aortic bypass via right thoracotomy without establishment of CPB. We believe such an approach is irrelevant for our patients because in every case there was a severe complication of disease and not only shunting but challenging resection of the aortic arch and isthmus was of demand. Moreover, esophagoplasty was performed in two cases. Safe and handy surgery was impossible via right thoracotomy without prior preparing of DAZ, stomach, and omental transplants.

Said et al 10 described ascending-to-descending posterior pericardial aortic bypass via sternotomy with usage of CPB and hypothermia. This technique is regarded unfit for the foregoing reasons: esophagoplasty is impossible via sternotomy (patients 1 and 2), and infected prosthesis should not be excised via the pericardium due to the risk of infection spread (patient 1). Pneumonectomy is possible via sternotomy. Nevertheless, simultaneous complex aortic resection was performed in all our patients. DAZ and thoracotomy provide simplicity of surgery and the best surgical control.

Coselli and LeMaire 11 described classical “clamp and sew” technique, combined with left heart bypass (LHB). LHB provides perfusion of a distal part of an aorta and decreases the risk of spinal and visceral ischemia. In our technique, this risk is minimal. On the one hand, we excise the proximal part of aorta only, but on the other hand, we never clamp it transversely when forming a DAZ.

Therefore the clear advantages, for example, universal technical simplicity in its every step, complete surgical control, convenience during all the surgery stages, and the possibility to solve the whole variety of concomitant medical problems (i.e., hemostasis, infection isolation, esophagoplasty, and bleeding prevention) allow stable long-term surgery outcomes. We consider the PSM to be a conceptually new approach to aortic arch and thoracic aorta surgery in the most severe complex diseases with a possible involvement of respiratory organs and the esophagus.
